# Characteristics and prognosis of rrDLBCL with *TP53* mutations and a high‐risk subgroup represented by the co‐mutations of *DDX3X‐TP53*

**DOI:** 10.1002/cam4.5756

**Published:** 2023-03-27

**Authors:** Fan Gao, Kai Hu, Peihao Zheng, Hui Shi, Xiaoyan Ke

**Affiliations:** ^1^ Department of Hematology The Second Affiliated Hospital of Xi'an Jiaotong University Xi'an China; ^2^ Department of Adult Lymphoma Beijing Boren Hospital Beijing China; ^3^ Department of Hematology Peking University Third Hospital Beijing China

**Keywords:** chimeric antigen receptor T‐cell therapy, *DDX3X* mutation, diffuse large B‐cell lymphoma, sequencing, *TP53* gene

## Abstract

**Background:**

TP53 mutations have a prognostic significance in relapsed and refractory diffuse large B‐cell lymphoma (rrDLBCL) patients, and their treatment still faces a great challenge. This study aimed to evaluate the prognosis of patients with TP53 mutations (TP53mut) in the context of CAR‐T therapy (Chimeric antigen receptor T‐cell therapy) as well as explore the heterogeneity in their cohort and identify the possible risk factors.

**Methods:**

A retrospective study was conducted to investigate the clinical characteristics of rrDLBCL patients with *TP53* mutations and their prognostic factors, receiving CAR‐T therapy. And the expression level of TP53 and DDX3X, which was an important co‐mutation of TP53 revealed in the cohort, were explored in public databases and cell lines.

**Results:**

The median overall survival time of 40 patients with *TP53* mutations was 24.5 months, while their median progression‐free survival time after CAR‐T was 6.8 months. There were no significant differences in the ORR (objective remission rate, *X* ^2^ = 3.0498, *p* > 0.05) and PFS (after CAR‐T therapy) between the patients with wild‐type and mutated *TP53* genes after CAR‐T therapy, while the OS of patients with *TP53* mutations was significantly worse (*p* < 0.01). In patients with *TP53* mutations, the performance status (ECOG score) was identified as the most important prognostic factor, while the efficacies of induction and salvage treatments were also correlated with the prognosis. Among molecular indicators, the co‐mutations of Chr‐17 and those located on the exon 5 of the *TP53* gene showed a tendency for a worse prognosis. Moreover, the patients with *TP53‐DDX3X* co‐mutations were identified as a subgroup with an extremely bad prognosis. The expression levels of *DDX3X* and *TP53* were explored in a public database and the cell lines with their co‐mutations, which indicated that inhibiting the *DDX3X* gene could affect the proliferation of rrDLBCL cells and the expression of TP53.

**Conclusions:**

This study indicated rrDLBCL patients with TP53 mutations was still the group of poor prognosis in the CAR‐T therapy era. CAR‐T therapy can benefit some TP53mut patients, and the performance status (ECOG) might help predict their prognosis. The study also revealed a subgroup of TP53‐DDX3X co‐mutations in rrDLBCL, which showed a strong clinical significance.

## Graphical abstract

A retrospective study was conducted to investigate the clinical characteristics of rrDLBCL patients with TP53 mutations and their prognostic factors, receiving CAR‐T therapy (chimeric antigen receptor T‐cell therapy), which revealed that the TP53 mutations still be a reverse prognostic factor. The median overall survival time of 40 patients with TP53 mutations was 24.5 months, while their median progression‐free survival time after CAR‐T was 6.8 months. In patients with TP53 mutations, the performance status (ECOG score) was identified as the most important prognostic factor. Moreover, the patients with TP53‐DDX3X co‐mutations were identified as a subgroup with an extremely bad prognosis.

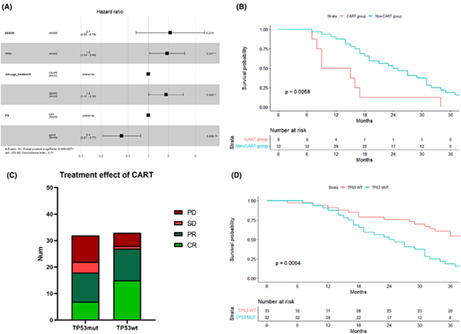

## 1 INTRODUCTION

*TP53* mutations, a long‐standing research hotspot, have been supported by abundant evidence for their prognostic significance in diffuse large B‐cell lymphoma (DLBCL).^1^, ^2^ However, the treatment of DLBCL with *TP53* mutations (TP53mut‐DLBCL) is still an unresolved problem. Based on the strong prognostic significance of these mutations in DLBCL, researchers suggest that it might be necessary to treat TP53mut‐DLBCL as a separate subtype.^3^, ^4^ In the past, due to the independence of *TP53* mutations and the poor prognosis of TP53mut patients, the significance of exploring intragroup heterogeneity was unclear. However, the emergence of novel therapies, such as Venetoclax and chimeric antigen receptor T‐cell therapy (CAR‐T therapy), has changed this concept.^5^ In particular, CAR‐T therapy has significantly improved the prognosis of relapsed and refractory DLBCL (rrDLBCL) with an objective remission rate (ORR) of more than 80%.^6^, ^7^ Numerous CAR‐T therapies have also been launched recently in China with an ORR reaching over 60%.^8^, ^9^ Therefore, the current study aimed to evaluate the prognosis of TP53mut patients in the context of CAR‐T therapy and to explore the heterogeneity in their cohort and identify the possible risk factors. In addition, an exploratory study was conducted on the TP53‐DDX3X (DEAD box protein 3, X‐chromosomal) co‐mutation subtype identified in this study.

## 2 MATERIALS AND METHODS

### 2.1 Patients

In this targeted sequencing retrospective study, the patients were recruited from a collaborative cohort of Peking University Third Hospital and Beijing Boren Hospital. The original cohort included patients with rrDLBCL and mediastinal large B‐cell lymphoma (PMBCL), and the study protocol was reviewed and approved by the ethics committees of the above‐mentioned two centers. In this study, the patients were further screened with the specified inclusion and exclusion criteria. The inclusion criteria were as follows: (1) the patients were diagnosed with DLBCL lymphoma at the Department of Pathology, Peking University Third Hospital using newly obtained biopsy specimens; (2) the patients were 18 years or older; and (3) the patients with rrDLBCL confirmed by two investigators (G.F and S.H) by reviewing the medical history and histopathological report. Relapsed DLBCL referred to the relapse condition after achieving complete response (CR) by the initial treatment. Refractory DLBCL was defined as the patients, receiving a standard induction regimen (R‐CHOP regimen) for at least four courses, did not achieve the complete clinical remission of lymph nodes and/or involved organs (Partial remission [PR] was not achieved), or new lesions appeared during treatment. The evaluation criteria were based on the 2014 Lugano criteria.^10^ The exclusion criteria were as follows: (1) the patients with Tly (transforming lymphoma) and PMBCL; (2) the patients, who failed to complete the induction regimen due to reasons other than disease progression; (3) among the patients with recurrence DLBCL cases, the samples obtained before recurrence were excluded, and those derived from the biopsy results of recurrence were included; and (4) the samples with double‐hit or triple‐hit lymphoma were excluded from the TP53mut‐related prognosis and survival analyses. The sequencing results of 96 patients were used for statistical analysis of mutation sites, while 83 patients, including 40 patients with *TP53* mutations, were screened for prognostic and survival analyses along with their follow‐up results.

## 3 METHODS OF TARGETED SEQUENCING

A core panel of 339 genes, which were selected based on their prior implications in the pathogenesis of hematologic diseases, was analyzed in rrDLBCL disease. The complete set of biotinylated long oligonucleotide probes to capture the coding exons of all the included 339 genes was purchased from Roche NimbleGen. The DNA libraries were generated using 225‐ng genomic DNA extracted from the frozen tumor tissues. Five 10‐μm‐thick sections per sample were used for the tumor DNA extraction using the QIAamp DNA Mini Kit following the manufacturer's instructions (Qiagen). The probe pool was hybridized to the tumor DNA (250 ng) upstream and downstream of each gene of interest. The pooled DNA libraries were loaded onto the cBot System for cluster generation followed by 2 × 150 paired‐end sequencing using the NextSeq550 sequencer (both from Illumina). The average depth of coverage across the targeted regions was approximately 3000 bp. The paired‐end sequencing reads, which were in FASTQ format, were mapped to the human genome (NCBI build 37) using BWA control software with default parameters. The mutational signatures were called using the output (BAM files) of the previous analysis with Vardict and MuTect2 software and stored as the final VCF file.

### 3.1 Cell lines

The human DLBCL cell lines, including OCI‐LY3, SU‐DHL‐6, OCI‐LY7, and WSU‐DLCL2 cell lines, were obtained from Meisen‐CTCC (Zhejiang Meisen Cell Technology Co., Ltd). After obtaining informed consent, the blood samples were obtained from healthy subjects in EDTA‐containing tubes, from which, the peripheral blood mononuclear cells (PBMCs) were isolated. The SU‐DHL‐6 and WSU‐DLCL2 cells were cultured in RPMI 1640 medium, containing 10% FBS, while the OCI‐LY3 and OCI‐LY7 cells were cultured in RPMI 1640 medium, containing 20% FBS (Gibco). All the cells were cultured at 37°C in a humidified chamber with a 5% CO_2_ concentration.

### 3.2 Real‐time quantitative PCR (RT‐qPCR)

The OCI‐LY7 and WSU‐DLCL2 cell lines were treated with RK‐33 (10 μM). After 24–48 h of treatment, the cells were harvested, and total RNA was extracted using TRIzol reagent (Invitrogen) and then reverse‐transcribed into cDNA using Hifair III 1st Strand cDNA Synthesis SuperMix for qPCR (YEASEN Biotech). The mRNA expression levels of target genes were determined using RT‐qPCR with Hieff qPCR SYBR Green Master Mix (YEASEN Biotech). The primer sequences for the RT‐qPCR were as follows: *DDX3X* forward, 5′‐AGCAGTTTTGGATCTCGTAGTG‐3′; *DDX3X* reverse, 5′‐ACTGTTTCCACCACGTTCAAAT‐3′; *TP53* forward, 5′‐ GAGGTTGGCTCTGACTGTACC‐3′; *TP53* reverse, 5′‐ TCCGTCCCAGTAGATTACCAC‐3′; *GAPDH* forward, 5′‐TCAAGGCTGAGAACGGGAAG‐3′; *GAPDH* reverse, 5′‐TCGCCCCACTTGATTTTGGA‐3′. The relative mRNA expression levels were calculated using the 2^−ΔΔCT^ method, and PBMCs were taken as negative control (NC).

### 3.3 Cell Counting Kit‐8 (CCK‐8) assay

Cell proliferation was determined using CCK‐8 reagent (Dojindo). After overnight incubation, the cells were seeded into 96‐well plates at a density of 5000 cells/well and treated with various concentrations of RK‐33 (1, 2.5, 5, 10, and 20 μM) followed by incubation at 37°C with 5% CO_2_ 0, 24, and 48 h. Then, the cells were incubated for 2 h with 10 μL of CCK‐8 solution. DMSO (dimethyl sulfoxide) was used as a control treatment. The optical density at 450 nm (OD450) values of each well was measured using a microplate reader.

### 3.4 Western blotting

Total cell protein was extracted using RIPA lysis buffer containing phosphatase inhibitor and protease inhibitor. Protein samples at an equal loading quantity were separated by 10% SDS‐PAGE before transferring onto nitrocellulose membranes for Western blotting. Transferred membranes were individually blocked in TBST containing 5% skimmed milk, followed by overnight incubation at 4°C with primary antibodies of p53 and β‐actin (Abcam); Immunodecorated membranes were washed with TBST buffer, followed by a 1‐hour incubation step at room temperature with anti‐mouse secondary antibodies and a TBST‐washing step afterward before fluorescent signal analysis using Odyssey infrared imaging system (LI‐COR).

### 3.5 Statistical analyses

Using the R language survival package (version 4.2.0), the Log‐Rank test and COX regression analysis were performed for the survival and multivariate analyses, respectively. Chi‐squared test and Fisher's exact test were used for the comparison of clinical information between the different subtypes, and a *t*‐test was used to compare the PCR (polymerase chain reaction) results using the R package. The visualization of relevant results was performed using the R package or GraphPad Prism 8.0.

## 4 RESULTS

### 4.1 Incidence

**FIGURE 1:**
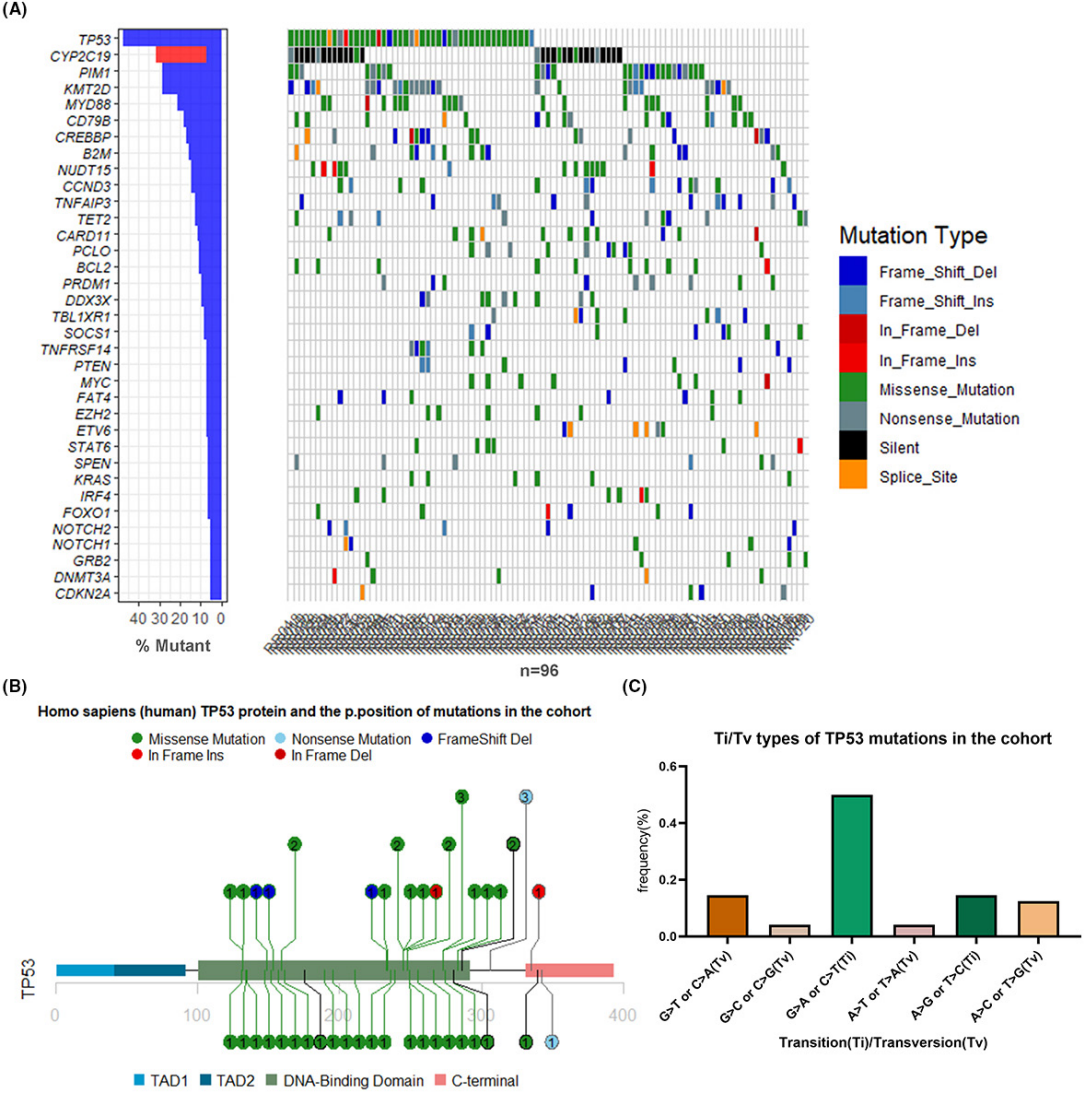
Heatmap of the mutations and *TP53* mutation hotspots in 96 Patients. (A) Waterfall plot of the major mutation frequencies in 96 patients. (B) Human TP53 protein and the amino acid positions of mutations in the cohort. (C) Ti/Tv distributions of *TP53* mutations in the cohort.

Among the total 96 patients, 46 patients carried *TP53* mutations, including 45 predicted deleterious *TP53* mutations (Combined Annotation Dependent Depletion [CADD] scores^11^ >10; 38 mutations had CADD scores >20). The complete follow‐up data were available for 85 of the total patients, as listed in Table 1, and the high‐frequency mutations in these patients are shown as a heatmap in Figure 1A. The main co‐mutations along with *TP53* mutations were present in the following genes: lysine‐specific methyltransferase 2D (*KMT2D*) (16/45, 35.6%), myeloid differentiation primary response 88 (*MYD88*) (11/45, 24.4%), beta‐2‐microglobulin (*B2M*) (9/45, 20%), Cyclic adenosine monophosphate Response Element Binding protein Binding Protein (*CREBBP*) (9/45, 20%), serine/threonine kinase Pim‐1 (*PIM1*) (8/45, 17.8%), *DDX3X* (6/45, 13.3%), nudix hydrolase 15 (*NUDT15*) (6/45, 13.3%), and TNF receptor superfamily member 14 (*TNFRSF14*) (6/45, 13.3%). However, after screening, only a few gene mutations showed limited correlations with *TP53* (support >0.05, confidence >0.1), such as *TNFRSF14* (lift = 1.81), *DDX3X* (lift = 1.40), and *B2M* (lift = 1.26), thereby reflecting the independence of *TP53*. See Data S1 for mutation information of 96 patients.

**TABLE 1:** Summary of the clinical information of the 96 rrDLBCL patients.

Characteristics	*n* (%)
Age (years)	
≤60	64 (66.6%)
>60	32 (33.3%)
Gender	
Male	49 (51.0%)
Female	47 (49.0%)
B symptom	
Yes	50 (52.1%)
No	46 (47.9%)
Pathological type (COO)	
GCB	30 (31.3%)
Non‐GCB	66 (68.7%)
Ann Arbor stage	
I	3 (3.1%)
II	8 (8.3%)
III	7 (7.3%)
IV	78 (81.3%)
IPI score	
0–1	8 (8.3%)
2	15 (15.6%)
3	31 (32.3%)
4–5	42 (43.8%)
Induction treatment	
CR/PR	61 (63.6%)
SD/PD	35 (36.5%)
Salvage treatment	
CR/PR	52 (54.2%)
SD/PD	44 (45.8%)

Analyzing the types of *TP53* mutations indicated that there were four deletion mutations, one insertion mutation, and 34 missense mutations in the exons 5 to 10. In addition, there were two nonsense mutations and three splice site mutations. Most of the mutations (40/45 88.9%) were present in the DNA‐binding domain of TP53; their specific location distribution is shown in Figure 1B. Among the 34 missense mutations, 15 mutations were transversions (Tv), while 19 mutations were transitions (Ti) **(**Data S2). The distribution of Ti/Tv types in the samples is presented in Figure 1C. Among these missense mutations, GC>AT (Ti), GC>TA (Tv), AT>GC (Ti), AT>CG (Tv), GC>CG (Tv), and AT>TA (Tv) were observed in 50%, 14.6%, 14.6%, 12.5%, 4.2%, and 4.2% of the samples, respectively. This distribution was similar to those observed in previous studies. Among all the 45 patients with *TP53* mutations, only seven patients had two different *TP53* mutations (Data S2).

Most of the mutations in the coding sequence of the *TP53* gene identified in the rrDLBCL patients in the current study had already been recorded in the UMD library^12^ and other previous *TP53* mutation databases,^13^ except for a few structural mutations, such as p.R335_E336insG, p.G245_M246delinsV, p.H233Lfs*14, and p.S149Yfs*20, etc. All these missense mutations had been predicted as harmful using numerous prediction tools, such as PolyPhen‐2 (Polymorphism Phenotyping v2), SIFT (Sorting Intolerant From Tolerant), Mut_ass (Mutationassessor), and PROVEAN (Protein Variation Effect Analyzer) (Data S2). In the current study, the CADD tool was used to screen the mutations with scores ≥10 for subsequent analysis. The amino acid positions 273 (three cases of p.R273H and one case of p.R273C) and 306 (three cases of p.R306X) were the most common mutation sites, which have also been previously identified as mutation hotspots.^14^ Other mutations, such as p.Y236D, p.T211P, and p.N131Y, had not been reported in lymphomas and were predicted as harmful using the above tools (Table S1). As its main co‐mutation, DDX3X showed no significant hot spot in the cohort, and the mutation sites of nine patients were different (p.T450S, p.T384A, p.K387X, and p.R528H, see Table S2).

### 4.2 Survival analysis of TP53mut patients

**FIGURE 2:**
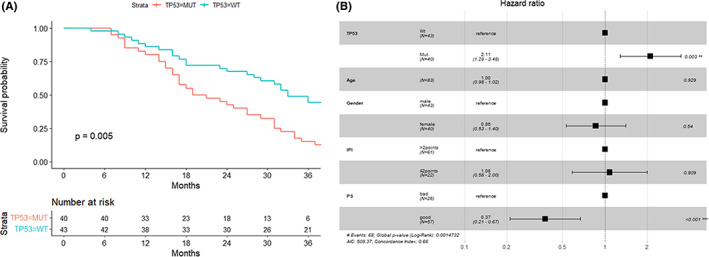
Prognostic significance of *TP53* mutations in the cohort of 83 patients. (A) OS analysis of the TP53mut and TP53wt patients in the whole cohort. (B) Multivariate COX regression analysis, including *TP53* mutations and other basic clinical variables.

The prognostic significance of *TP53* mutations for the entire rrDLBCL cohort (*n* = 83) was evaluated. Among the patients included for survival analysis, the median follow‐up time was 3.54 years, and the 2‐year survival rate was 45% (18/40). The patients with *TP53* mutations (TP53mut, *n* = 40) had significantly worse prognoses as compared to those with wild‐type *TP53* (TP53wt, *n* = 43) (*p* < 0.01, Figure 2A). In the multivariate analysis, including included basic clinical variables, the *TP53* mutations (HR = 2.11, *p* < 0.01) and good performance status (ECOG score <2, HR = 0.37, *p* < 0.01) were independent prognostic factors, and the global *p*‐value of the model was less than 0.01 (Figure 2B). Only the *TP53* mutations (Data S2) showed significant prognostic significance (*p* < 0.01) in multivariate analysis among the high‐frequency mutations (>15%). The clinical information of the 40 patients with *TP53* mutations is listed in Table 2.

**TABLE 2:** Summary of the clinical information of 40 rrDLBCL patients with *TP53* mutations.

	TP53mut patients (*n* = 40)		
	CAR‐T group	non‐CAR‐T group	*p*‐value
Num (*n*, %)	32 (80.0)	8 (20.0)	
Age (mean, SD)	48.7 (13.0)	49.5 (16.6)	
Gender (*n*, %)			
Male	19 (59.4)	4 (50.0)	0.702
Female	13 (40.6)	4 (50.0)	
Pathological subtype (*n*, %)			
GCB	19 (59.4)	2 (25.0)	0.686
Non‐GCB	13 (40.6)	6 (75.0)	
Performance status (*n*, %)			
ECOG<2	21 (65.6)	6 (75.0)	1
ECOG≥2	11 (34.4)	2 (25.0)	
Ann arbor stage (*n*, %)			
I–II	1 (3.1)	2 (25.0)	0.13
III	2 (6.3)	0 (0.0)	
IV	29 (90.6)	6 (75.0)	
IPI score (*n*, %)			
0–2 points	5 (15.6)	3 (37.5)	0.32
3–5 points	27 (84.4)	5 (62.5)	
Induction treatment (*n*, %)			
CR/PR	20 (62.5)	4 (50.0)	0.691
SD/PD	12 (37.5)	4 (50.0)	
Salvage treatment (*n*, %)			
CR/PR	13 (40.6)	3 (37.5)	1
SD/PD	19 (59.4)	5 (62.5)	
Co‐mutations (*n*, %)			
PIM1	8 (25.0)	0 (0.0)	0.17
MYD88	7 (21.9)	2 (25.0)	1
CD79B	5 (15.6)	0 (0.0)	0.56
DDX3X	4 (12.5)	2 (25.0)	0.58
Median overall survival (month)	24.5	12	0.01
Median progression‐free survival (After first CAR‐T therapy, month)	6.8	NA	
2‐year survival rate (%)	53.1	14.3	0.05

**FIGURE 3:**
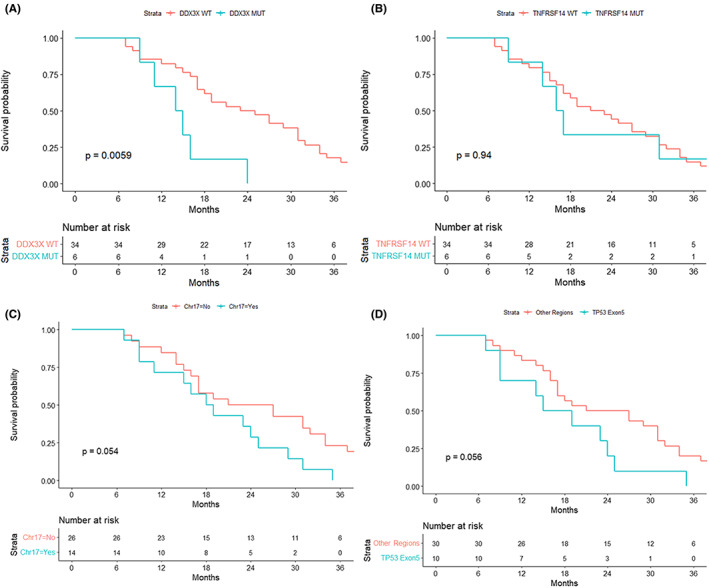
Significance of molecular biological variables in the patients with *TP53* mutations. (A) OS analysis of DDX3X co‐mutations in the patients with *TP53* mutations. (B) OS analysis of *TNFRSF14* co‐mutations in the patients with *TP53* mutations. (c) OS analysis of Chr17 co‐mutations in the patients with *TP53* mutations. d. OS analysis of *TP53* mutations located in exon 5.

Among the two major co‐mutations, the patients with DDX3Xmut and TP53mut co‐mutations showed a very poor prognosis (6/40, 15%, 5 of GCB, and 1 of Non‐GCB), which was significantly different from that of the patients with TP53mut‐DDX3Xwt (*p* < 0.01, Figure 3A). On the contrary, *TNFRSF14* mutations had no significant effect on the prognosis of patients with *TP53* (*p* > 0.1, Figure 3B). Similarly, the other high‐frequency co‐mutations, such as *KMT2D*, *CREBBP*, and *MYD88* mutations, did not show significant prognostic significance. Among the patients with *TP53* mutations, about one‐third of the patients had co‐mutations located on chromosome 17, including *CD79B* (cluster of differentiation 79B), *GNA13* (G protein subunit alpha 13), and *STAT3* (signal transducer and activator of transcription 3) genes. These patients showed a worse prognosis (*p* = 0.054, Figure 3C). The patients with mutations located in the exon 5 of the *TP53* gene also showed a worse prognosis as compared to those with mutations in other regions (*p* = 0.056, Figure 3D).

**FIGURE 4:**
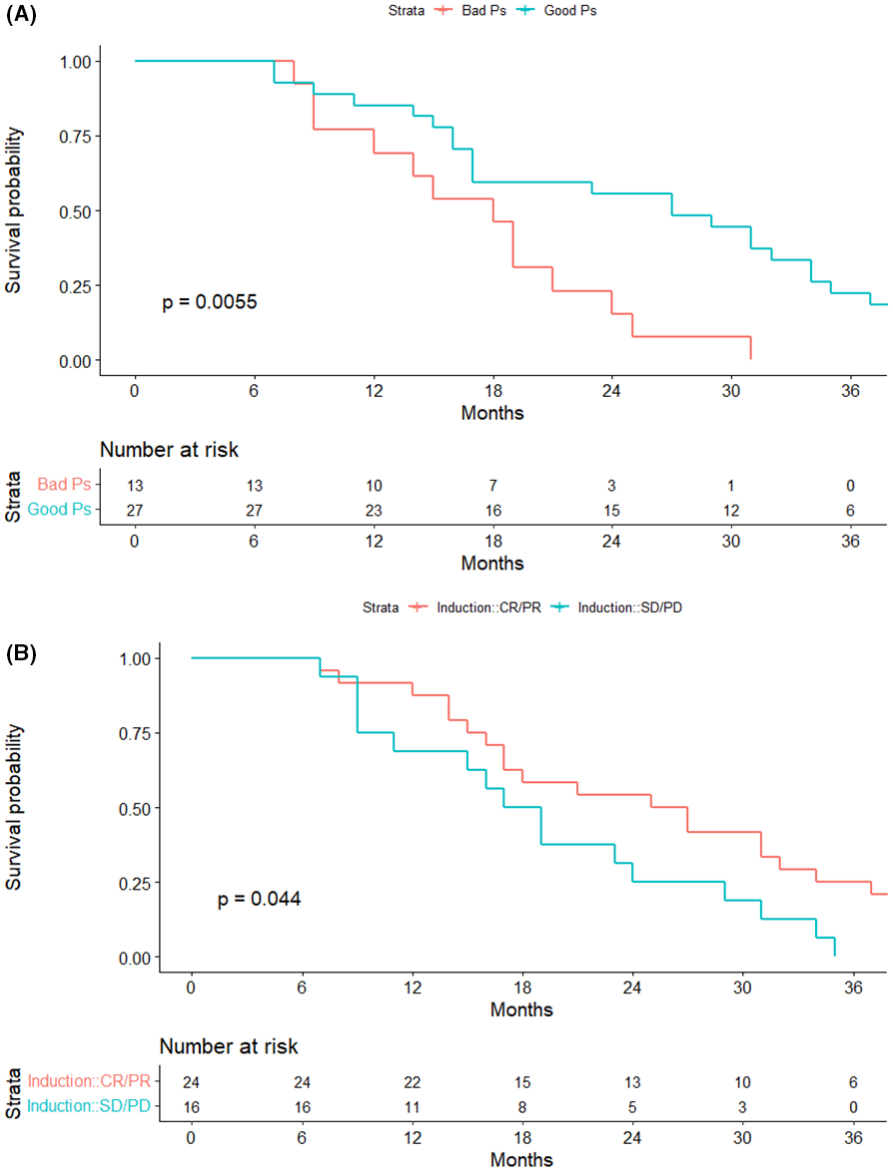
Significance of clinical variables in the patients with *TP53* mutations. (A) OS analysis between the different groups of induction therapy efficacy. (B) OS analysis of patients between the different groups of performance status (ECOG scores).

Among the main clinical indicators, the performance status (ECOG score) (*p* < 0.05, Figure 4A), the efficacy of induction therapy (*p* < 0.05, Figure 4B), and the efficacy of salvage therapy (*p* < 0.05) could significantly predict the prognosis of patients with *TP53* mutations. The COX regression analysis of the performance status (ECOG), induction therapy efficacy, and DDX3X and Chr17 co‐mutations also showed that these factors could still significantly predict the prognosis of patients with *TP53* mutations (ECOG score <2, HR = 0.41, *p* < 0.05, Figure S1).

### 4.3 Efficacy of CAR‐T therapy in patients with *TP53* mutations

**FIGURE 5:**
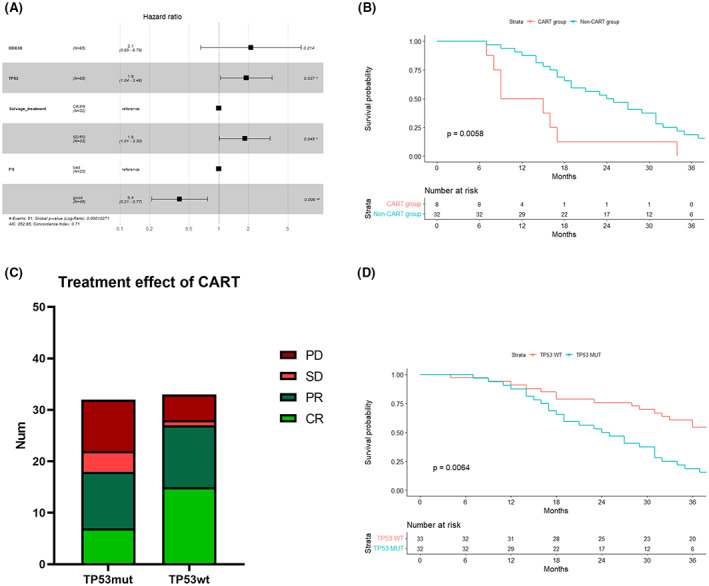
Evaluation of the treatment effects of CAR‐T therapy in patients with *TP53* mutations. (A) COX regression multivariate analysis of the patients receiving CAR‐T therapy. (B) OS analysis of the patients with *TP53* mutations in the CAR‐T/non‐CAR‐T groups. (C) Histogram of CAR‐T therapy efficacy in the patients with wild‐type and mutated *TP53* gene. (D) OS analysis of the patients with wild‐type and mutated *TP53* gene, receiving CAR‐T therapy.

In this study, 65 patients, receiving CAR‐T therapy, were followed up. For these patients, the *TP53* mutations (*p* < 0.01), *DDX3X* mutations (*p* < 0.001), performance status (*p* < 0.01), and salvage treatment effect (*p* < 0.01) had significant prognostic significance. Among the CAR‐T therapy‐receiving patients, there were only four patients with *DDX3X* mutations, and no one among them achieved PR, and their median survival time was only 12.5 months. The multivariate analysis of CAR‐T therapy‐receiving patients also showed that the *TP53* mutations (HR = 1.9, *p* < 0.05), performance status (ECOG score <2, HR = 0.4, *p* < 0.01), and salvage treatment effect (HR = 1.8, *p* < 0.05) were still the most important predictors of OS in the CAR‐T therapy‐receiving patients (Figure 5A). These variables were also the best predictors of achieving CR by CAR‐T therapy.

A total of 80% (32/40) of patients with *TP53* mutations received CAR‐T therapy. The median OS of patients with *TP53* mutations was 24.5 months, and the median PFS after CAR‐T therapy was 6.8 months. Although CAR‐T therapy significantly improved the prognosis of patients with *TP53* mutations as compared to those who did not receive CAR‐T therapy (*p* < 0.01, Figure 5B), the overall ORR was only 56.2% (18/32), which was slightly lower than the ORR for the entire CAR‐T therapy‐receiving cohort (45/65, 69.2%). The ORR of the patients with wild‐type *TP53* was 81.8% (27/33) as compared to those receiving CAR‐T therapy. There was no significant difference in ORR (*χ* ^2^ = 3.0498, *p* > 0.05, Figure 5C) and PFS (after CAR‐T therapy, *p* > 0.1) between the patients with wild‐type and mutated *TP53* gene. However, there was still a significant difference in the OS times of the patients between the two groups, showing a worse OS of the patients with *TP53* mutations in the CAR‐T therapy‐receiving cohort (*p* < 0.01, Figure 5D). Seven patients with *TP53* mutations achieved CR (7/32, 21.9%) with a median OS time of 35 months and a median PFS time of 30 months after CAR‐T therapy. Nine patients, who did not achieve PR in salvage treatment, finally achieved remission through CAR‐T therapy and accounted for 47.4% (9/19) of the patients, showing salvage treatment failure (SD or PD).

### 4.4 Recurrent *TP53*‐*DDX3X* co‐mutations and their expression levels in cell lines

The possible adverse prognostic effects of *DDX3X* mutations have already been discussed in the previous sections. In this study cohort, there were only nine patients with *DDX3X* mutations, and their median survival time was only 15.27 months. Six of the nine patients had *TP53* co‐mutations and could not get PR from salvage therapy, including CAR‐T therapy. The *DDX3X* mutations were identified in the Sanger sequencing results provided by the Cell Model Passport:^15^ A total of 23 cell lines in the database carried *DDX3X* mutations (seven of 23 cells lines were the cell lines of B‐Cell Non‐Hodgkin's Lymphoma, and five were the cell lines of Burkitt's Lymphoma). Among these 23 cell lines, 19 cell lines also carried *TP53* mutations (19/23, 82.6%), suggesting the recurrence of *DDX3X*‐*TP53* co‐mutations.

**FIGURE 6:**
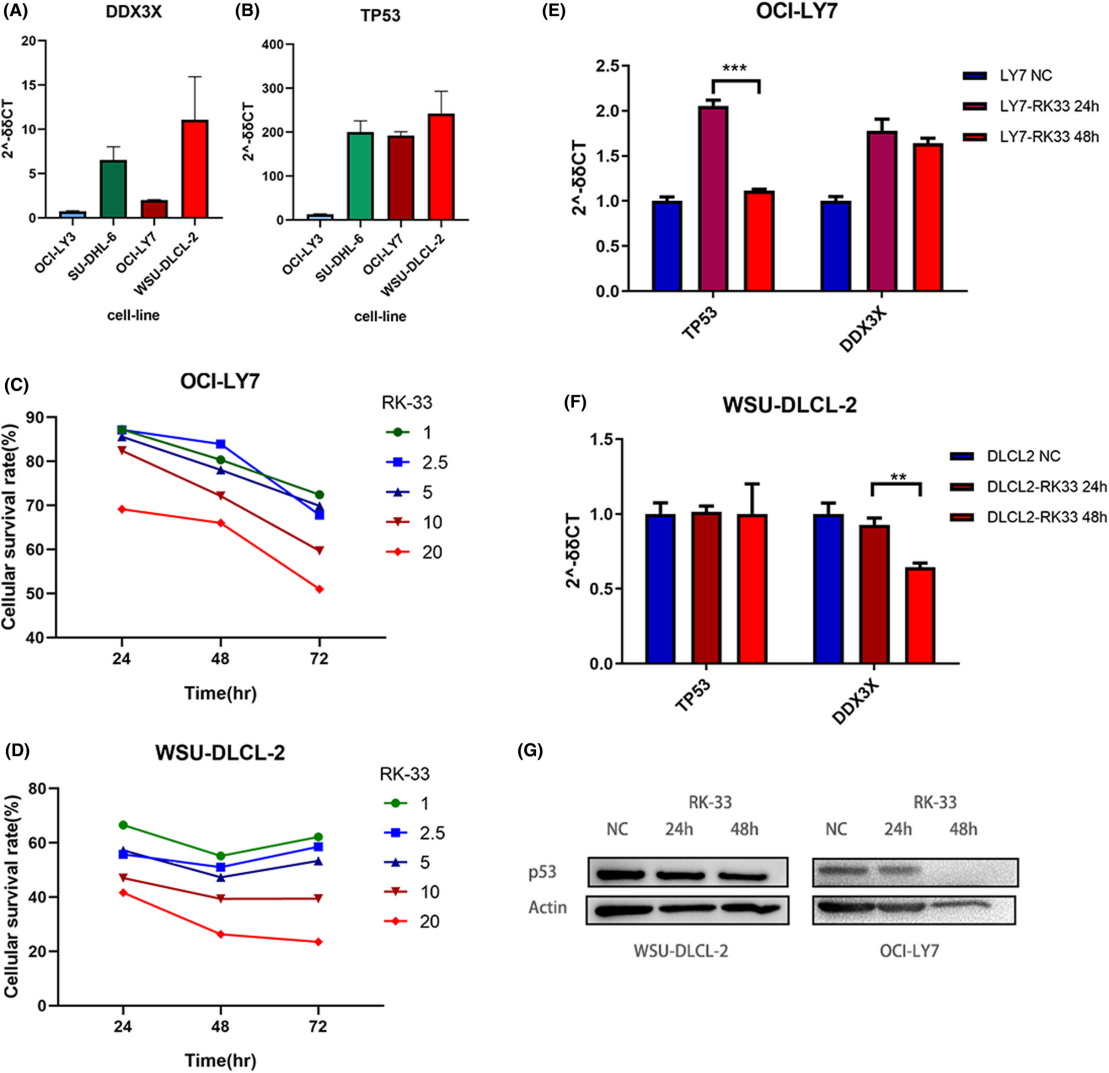
Expression levels of *DDX3X* and *TP53* in DLBCL cell lines and CCK‐8 cell proliferation test results. (A) Expression levels of *DDX3X* were analyzed using RT‐PCR in DLBCL cell lines. (B) Expression levels of *TP53* were analyzed using RT‐PCR in DLBCL cell lines. (C) Inhibitory effects of RK‐33 on the cell proliferation of OCI‐LY7 cell line analyzed using CCK8 assay. (D) Inhibitory effects of RK‐33 on the cell proliferation of WSU‐DLCL‐2 cell line analyzed using CCK8 assay. (E) Inhibitory effects of RK‐33 on the expression levels of *DDX3X* and *TP53* in the OCI‐LY7 cell lines. (F) Inhibitory effects of RK‐33 on the expression levels of *DDX3X* and *TP53* in the WSU‐DLCL‐2 cell lines. (G) Western blot results of p53 with RK‐33 intervention in WSU‐DLCL‐2 and OCI‐LY7 cell lines.

In the cell lines of B‐cell non‐Hodgkin's lymphoma and Burkitt's lymphoma (*n* = 63), the RNA‐Seq read counts of *DDX3X* and *TP53* were positively correlated (PCCs [Pearson correlation coefficient] = 0.49, *p* < 0.0001, Figure S2a). The correlations between expression level of *TP53* and *DDX3X* in a cohort of DLBCL patients were further validated. In both the large‐scale DLBCL cohorts of GSE10846 and GSE31312 datasets, the *TP53* and *DDX3X* RNA‐Seq read counts showed strong correlations (*p* < 0.0001, PCCs = 0.27–0.32, Figure S2b,c). The expression levels of DDX3X were higher €n the two *DDX3X*‐mutant cell lines as compared to those in OCI‐LY3 (DDX3Xwt‐TP53wt); however, the expression level of *DDX3X* in SU‐DHL‐6 cell line (TP53mut‐DDX3Xwt) was also high (Figure 6A,B). The CCK‐8 analysis showed that the *DDX3X* inhibitor (RK‐33^16^, ^17^) could inhibit the proliferation of *DDX3X*‐mutant cell lines of OCI‐LY7 and WSU‐DLCL‐2, especially that of OCI‐LY7 cell line (Figure 6C,D). RT‐PCR analysis showed that the expression level of *TP53* in the OCI‐LY7 cell line decreased significantly after 48 h of RK‐33 treatment (*p* < 0.001); However, this phenomenon was not observed in the WSU‐DLCL‐2 cell line (Figure 6E,F). In the Western blot experiment, OCI‐LY7 also responded more obviously to RK‐33, and p53 showed a downward trend after adding inhibitor for 24–48 h (Figure 6G).

## 5 DISCUSSION

In this study, the heterogeneity of prognosis in the rrDLBCL patient with *TP53* mutations in the context of CAR‐T therapy was explored. The follow‐up data showed that the CAR‐T therapy had a considerable effect on the rrDLBCL with *TP53* mutations, about half of the patients, who did not achieve remission in salvage treatment, benefited from CAR‐T therapy. The CAR‐T thereby significantly improved the prognosis of patients with CR; however, these patients accounted for only about 1/5 of all the patients with *TP53* mutations. The OS of patients with *TP53* mutations in the CAR‐T therapy cohort was poor, and their ORR was also lower than that of the patients with wild‐type *TP53*. This study indicated that CAR‐T therapy might not be the final solution for patients with *TP53*; however, it might act as a “relay statio” with great potential. Only eight patients in this cohort did not receive CAR‐T therapy, which might bring bias and cause difficulties in interpretation. Therefore, the results of a subgroup study based on molecular biology in a large‐scale prospective study should be performed in the future.

The results showed that performance status was the most important prognostic factor among the clinical indicators, and the efficacy of front‐line treatment showed prognostic significance as well. The analysis of clinical variables suggested that the impact of performance status on the prognosis of patients with *TP53* mutations before CAR‐T therapy should be fully considered. Among the molecular biological markers, the status of *DDX3X* and Chr17 co‐mutations might affect the prognosis of rrDLBCL patients with *TP53* mutations. The Chr17 co‐mutations included the mutations in *CD79B*, *GNA13*, and *STAT3* genes, which have been proven to be correlated with the prognosis of DLBCL in a previous study.^18^ It was speculated that *CD79B* might play a certain role in prognosis, thereby affecting the prognosis analysis results of the Chr17 group. First, the *CD79B* mutations were the most common (5/14, 35.7%) mutations among Chr17 co‐mutations. Second, the patients with *CD79B* and *TP53* co‐mutations showed a tendency to relapse or progress earlier after the CAR‐T therapy (PFS after CAR‐T therapy, *p*‐value of K–M survival analysis was <0.05). The different *TP53* mutation sites were generally discrete in the cohort, and it was difficult to analyze the prognostic significance of each site. Only the mutations on exon 5 showed a tendency of poor prognosis; however, the tendency was statistically insignificant (*p* = 0.056).

DDX3X is a ubiquitously expressed RNA helicase, which is involved in the multiple stages of RNA biogenesis. It has about 34%, and 15% incidence in Burkitt lymphoma and adult Burkitt lymphoma, respectively.^19^ A study showed that the loss‐of‐function mutations in *DDX3X* were also enriched in the MYC‐translocated DLBCL and revealed functional correlations between mutant *DDX3X* and *MYC*.^20^ Researchers suggested that this was relatively common in the so‐called molecular high‐grade B‐Cell Lymphoma among DLBCL patients.^21^ As sex chromosome‐specific genes, the functional difference between *DDX3X* and *DDX3Y* might explain the gender differences in the incidence rate of Burkitt lymphoma.^22^ However, in this study, the proportion of men and women with *DDX3X* mutations was almost similar (5/4, 1.25: 1). All the patients with *DDX3X* mutations showed poor prognosis; only 2 of them had MYC split/rearrangement. Since the patients with double‐hit and triple‐hit lymphomas were excluded from this study, it was speculated that identifying the *DDX3X* mutations at the time of relapse might be valuable for evaluating the prognosis of rrDLBCL patients. The prognostic potential of *DDX3X* mutations has also been emphasized in previous studies;^23^ however, the studies conducted on the response of patients with *DDX3X* mutations to CAR‐T therapy are still lacking. The current study suggested that *DDX3X* had a prominent prognostic significance in rrDLBCL, and the patients with *DDX3X* mutations might not benefit from salvage treatments, including CAR‐T therapy. It is worth mentioning that the frequency of co‐mutation of TP53‐DDX3X reported in this study reached 13.3% in the original cohort (*n* = 96, and 15% in the cohort for survival analysis), which might be overestimated. Because the prognosis of patients in the cohort was generally poor, and only two centers participated in the study, which could bring deviation.

The expression levels of *DDX3X* in different tumors are inconsistent.^24^ Even in the published studies on DLBCL, there are contrasting results about the function of *DDX3X*, indicating its complexity. Kizhakeyil et al. established the *DDX3X*‐mutant and *DDX3X*‐knockout cell lines to observe their effects on the proliferation, apoptosis, and other phenotypes of the cell lines. The results showed that the overexpression of wild‐type *DDX3X* in the U2932 and HuT78 cells significantly decreased their proliferation.^23^ Lacroix et al revealed that DDX3X was required for the lymphoid differentiation and MYC‐driven lymphomagenesis, which indicated that inhibiting the expression of the *DDX3X* gene could be a treatment strategy for the MYC‐driven B‐cell lymphoma.^25^ The current study analyzed the expression levels of *DDX3X* in different DLBCL cell lines using RT‐PCR. As compared to the cell lines with the wild‐type *DDX3X* gene (OCI‐LY3/SU‐DHL‐6), those with the mutated *DDX3X* gene (OCI‐LY7/WSU‐DLCL‐2) showed an upregulated expression level of *DDX3X*. On the contrary, the expression level of *DDX3X* in the cell lines with mutated *TP53* gene (SU‐DHL‐6/OCI‐LY7/WSU‐DLCL2) was upregulated, suggesting that the expression of *TP53* might affect that of *DDX3X*. Therefore, more samples from real patients are still needed to observe the specific phenotypic effects of *DDX3X* mutations in DLBCL. DDX3X can bind to both the wild‐type and mutant TP53 proteins in tumors. When DNA damage occurs, DDX3X can still bind to the wild‐type TP53 and stabilize its protein level, thereby promoting TP53‐mediated apoptosis.^24^ At the same time, a study suggested that TP53 might directly regulate the transcription of the *DDX3X* gene in lung cancer, and the relevant research results also supported the existence of the TP53‐DDX3X pathway.^26^ In this study, both the cell model database and the clinical cohort identified the *TP53*‐*DDX3X* co‐mutations as a recurrent phenomenon. The CCK‐8 assay indicated that the inhibitor of DDX3X (RK‐33) decreased the proliferation of DLBCL cell lines having *TP53*‐*DDX3X* co‐mutations. However, the response to RK‐33 at the expression level was only observed in the OCI‐LY7 cell line, showing the downregulation of the *TP53* expression level, which could be purely due to off‐target toxicity of RK‐33. At present, the characteristics of DDX3X in lymphoma are still very mysterious and have not been fully revealed. The current experimental results are not enough to answer questions about it. Our research team will conduct more studies to evaluate the functional correlations between the two mutations in the future.

## 6 CONCLUSIONS

Overall, this study investigated the clinical characteristics of rrDLBCL patients with *TP53* mutations in the context of CAR‐T therapy, which was still the group of patients with poor prognosis in the CAR‐T therapy era. CAR‐T therapy can benefit some patients with *TP53* mutations, and the performance status (ECOG), an important clinical indicator, might help predict their prognosis. At the same time, this study also revealed a subgroup of *TP53‐DDX3X* co‐mutations in rrDLBCL, which showed a strong clinical significance. Although these co‐mutations were uncommon in DLBCL, their response to treatment was extremely poor, and even CAR‐T therapy could not reverse their prognosis. Therefore, it might be very necessary to detect *DDX3X* mutations when the disease relapses/progresses. Furthermore, as a potential target for *TP53* regulation, *DDX3X* might have a promising potential in cancer research, and its studies might bring benefits to DLBCL patients.

## AUTHOR CONTRIBUTIONS

**Fan Gao:** Conceptualization (lead); data curation (equal); formal analysis (lead); investigation (lead); methodology (equal); software (lead); visualization (lead); writing – original draft (lead); writing – review and editing (equal). **Kai Hu:** Resources (equal); validation (equal); writing – review and editing (equal). **Peihao Zheng:** Data curation (equal); investigation (equal). **Hui Shi:** Data curation (equal); validation (equal). **Xiaoyan Ke:** Project administration (lead); resources (lead); supervision (lead).

## FUNDING INFORMATION

No funding.

## CONFLICT OF INTEREST STATEMENT

The authors have no conflict of interest.

## ETHICS STATEMENT

Authors must declare all information about ethics in this section including followings as appropriate: Approval of the research protocol by an Institutional Reviewer Board: The studies involving human participants were reviewed and approved by the Peking University Third Hospital. Informed Consent: N/A. Registry and the Registration No. of the study/trial: N/A. Animal Studies: N/A.

## Supporting information


Data S1

Data S2

Data S3

Figure S1

Figure S2

Table S1

Table S2

## Data Availability

The original contributions presented in the study are included in the article/Supplementary Material, further inquiries can be directed to the corresponding author.

## References

[1] Zenz T , Kreuz M , Fuge M , et al. TP53 mutation and survival in aggressive B cell lymphoma. Int J Cancer. 2017;141(7):1381‐1388. 28614910 10.1002/ijc.30838

[2] Voropaeva EN , Pospelova TI , Voevoda MI , Maksimov VN , Orlov YL , Seregina OB . Clinical aspects of *TP53* gene inactivation in diffuse large B‐cell lymphoma. BMC Med Genomics. 2019;12(Suppl 2):35. 30871527 10.1186/s12920-019-0484-9 PMC6416833

[3] Wright GW , Huang DW , Phelan JD , et al. A probabilistic classification tool for genetic subtypes of diffuse large B cell lymphoma with therapeutic implications. Cancer Cell. 2020;37(4):551‐568.e14. 32289277 10.1016/j.ccell.2020.03.015 PMC8459709

[4] Gao F , Tian L , Shi H , et al. Genetic landscape of relapsed and refractory diffuse large B‐cell lymphoma: a systemic review and association analysis with next‐generation sequencing. Front Genet. 2021;12:677650. 34925435 10.3389/fgene.2021.677650 PMC8675234

[5] Deng M , Xu‐Monette ZY , Pham LV , et al. Aggressive B‐cell lymphoma with MYC/TP53 dual alterations displays distinct clinicopathobiological features and response to novel targeted agents. Mol Cancer Res. 2021;19(2):249‐260. 33154093 10.1158/1541-7786.MCR-20-0466 PMC8092941

[6] Abramson JS , Johnston PB , Kamdar M , et al. Health‐related quality of life with lisocabtagene maraleucel vs standard of care in relapsed or refractory LBCL. Blood Adv. 2022;6(23):5969‐5979. 36149968 10.1182/bloodadvances.2022008106 PMC9713278

[7] Locke FL , Ghobadi A , Jacobson CA , et al. Long‐term safety and activity of axicabtagene ciloleucel in refractory large B‐cell lymphoma (ZUMA‐1): a single‐arm, multicentre, phase 1‐2 trial. Lancet Oncol. 2019;20(1):31‐42. 30518502 10.1016/S1470-2045(18)30864-7 PMC6733402

[8] Ying Z , Yang H , Guo Y , et al. Relmacabtagene autoleucel (relma‐cel) CD19 CAR‐T therapy for adults with heavily pretreated relapsed/refractory large B‐cell lymphoma in China. Cancer Med. 2021;10(3):999‐1011. 33382529 10.1002/cam4.3686 PMC7897944

[9] Shi H , Zheng P , Liu R , et al. Genetic landscapes and curative effect of CAR T‐cell immunotherapy in relapse and refractory DLBCL patients. Blood Adv. 2022;bloodadvances.2021006845. 10.1182/bloodadvances.2021006845 PMC10034568 35901280

[10] Cheson BD , Fisher RI , Barrington SF , et al. Recommendations for initial evaluation, staging, and response assessment of Hodgkin and non‐Hodgkin lymphoma: the Lugano classification. J Clin Oncol. 2014;32(27):3059‐3068. 25113753 10.1200/JCO.2013.54.8800 PMC4979083

[11] Rentzsch P , Schubach M , Shendure J , Kircher M . CADD‐splice‐improving genome‐wide variant effect prediction using deep learning‐derived splice scores. Genome Med. 2021;13(1):31. 33618777 10.1186/s13073-021-00835-9 PMC7901104

[12] Leroy B , Ballinger ML , Baran‐Marszak F , et al. Recommended guidelines for validation, quality control, and reporting of TP53 variants in clinical practice. Cancer Res. 2017;77(6):1250‐1260. 28254861 10.1158/0008-5472.CAN-16-2179 PMC7457206

[13] Edlund K , Larsson O , Ameur A , et al. Data‐driven unbiased curation of the *TP53* tumor suppressor gene mutation database and validation by ultradeep sequencing of human tumors. Proc Natl Acad Sci USA. 2012;109(24):9551‐9556. 22628563 10.1073/pnas.1200019109 PMC3386058

[14] Hu J , Cao J , Topatana W , et al. Targeting mutant p53 for cancer therapy: direct and indirect strategies. J Hematol Oncol. 2021;14(1):157. 34583722 10.1186/s13045-021-01169-0 PMC8480024

[15] van der Meer D , Barthorpe S , Yang W , et al. Cell Model Passports‐a hub for clinical, genetic and functional datasets of preclinical cancer models. Nucleic Acids Res. 2019;47(D1):D923‐D929. 30260411 10.1093/nar/gky872 PMC6324059

[16] Yang SNY , Atkinson SC , Audsley MD , Heaton SM , Jans DA , Borg NA . RK‐33 is a broad‐spectrum antiviral agent that targets DEAD‐box RNA helicase DDX3X. Cell. 2020;9(1):170. 10.3390/cells9010170 PMC7016805 31936642

[17] Tantravedi S , Vesuna F , Winnard PT , et al. Targeting DDX3 in medulloblastoma using the small molecule inhibitor RK‐33. Transl Oncol. 2019;12(1):96‐105. 30292066 10.1016/j.tranon.2018.09.002 PMC6171097

[18] Dubois S , Viailly PJ , Mareschal S , et al. Next‐generation sequencing in diffuse large B‐cell lymphoma highlights molecular divergence and therapeutic opportunities: a LYSA study. Clin Cancer Res. 2016;22(12):2919‐2928. 26819451 10.1158/1078-0432.CCR-15-2305

[19] Burkhardt B , Michgehl U , Rohde J , et al. Clinical relevance of molecular characteristics in Burkitt lymphoma differs according to age. Nat Commun. 2022;13(1):3881. 35794096 10.1038/s41467-022-31355-8 PMC9259584

[20] Gong C , Krupka JA , Gao J , et al. Sequential inverse dysregulation of the RNA helicases DDX3X and DDX3Y facilitates MYC‐driven lymphomagenesis. Mol Cell. 2021;81(19):4059‐4075.e11. 34437837 10.1016/j.molcel.2021.07.041

[21] Sha C , Barrans S , Cucco F , et al. Molecular high‐grade B‐cell lymphoma: defining a poor‐risk group that requires different approaches to therapy. J Clin Oncol. 2019;37(3):202‐212. 30523719 10.1200/JCO.18.01314 PMC6338391

[22] Shen H , Yanas A , Owens MC , et al. Sexually dimorphic RNA helicases DDX3X and DDX3Y differentially regulate RNA metabolism through phase separation. Mol Cell. 2022;82(14):2588‐2603.e9. 35588748 10.1016/j.molcel.2022.04.022 PMC9308757

[23] Kizhakeyil A , Mohammed Zaini NB , Poh ZS , et al. DDX3X loss is an adverse prognostic marker in diffuse large B‐cell lymphoma and is associated with chemoresistance in aggressive non‐Hodgkin lymphoma subtypes. Mol Cancer. 2021;20(1):134. 34654425 10.1186/s12943-021-01437-0 PMC8520256

[24] Mo J , Liang H , Su C , Li P , Chen J , Zhang B . DDX3X: structure, physiologic functions and cancer. Mol Cancer. 2021;20(1):38. 33627125 10.1186/s12943-021-01325-7 PMC7903766

[25] Lacroix M , Beauchemin H , Fraszczak J , et al. The X‐linked helicase DDX3X is required for lymphoid differentiation and MYC‐driven lymphomagenesis. Cancer Res. 2022;82(17):3172‐3186. 35815807 10.1158/0008-5472.CAN-21-2454

[26] Wu DW , Liu WS , Wang J , Chen CY , Cheng YW , Lee H . Reduced p21^WAF1/CIP1^ via alteration of p53‐DDX3 pathway is associated with poor relapse‐free survival in early‐stage human papillomavirus‐associated lung cancer. Clin Cancer Res. 2011;17(7):1895‐1905. 21325288 10.1158/1078-0432.CCR-10-2316

