# Supplementary material for: Characteristics and prognosis of rrDLBCL with TP53 mutations and a high‐risk subgroup represented by the co‐mutations of DDX3X‐TP53
Source: Cancer Med. 2023 Mar 27;12(9):10267–79. doi: 10.1002/cam4.5756 (PMC10225230; doi:10.1002/cam4.5756)
Supplement: Supplementary file 3 — Data S3 [file CAM4-12-10267-s005.pdf]

ID	Sample_number	CART_therapy	Age	Gender	COO	Ann_arbor_stage	B_symptom	ECOG	IPI	Induction	Salvage	OS(m)	OS_Statue
RR001	DL55	Yes	64	male	non_GCB	IV	B	4	5	PD	PD	24	dead
RR002	DL88	Yes	76	female	non_GCB	IV	B	1	4	CR	PR	43	dead
RR003	DL56	Yes	65	female	non_GCB	IV	A	2	4	PR	PD	41	dead
RR005	DL57	Yes	45	female	non_GCB	IV	B	3	4	CR	CR	55	survive
RR006	DL51	Yes	37	male	non_GCB	IV	A	2	4	PR	PD	12	dead
RR007	DL40	Yes	37	male	non_GCB	IV	A	1	3	CR	PR	83	survive
RR008	DL53	Yes	41	male	non_GCB	III	A	1	2	SD	PD	38	dead
RR009	DL49	Yes	47	female	non_GCB	IV	B	1	1	PR	PR	46	survive
RR076	DL54	Yes	54	female	non_GCB	IV	B	1	3	PD	CR	44	dead
RR010	DL41	Yes	49	male	GCB	IV	A	1	2	PD	PD	33	dead
RR011	DL20	Yes	54	male	non_GCB	IV	B	3	4	PD	PD	19	dead
RR012	DL31	Yes	43	male	GCB	IV	B	3	4	PD	SD	9	dead
RR014	DL34	No	79	male	non_GCB	IV	A	2	5	PR	PR	17	dead
RR015	DL58	No	47	male	GCB	IV	B	3	4	PD	PR	32	dead
RR016	DL59	Yes	56	female	non_GCB	IV	B	2	4	PR	CR	na	na
RR017	DL94	No	62	female	GCB	IV	A	3	4	CR	PD	11	dead
RR018	DL38	No	65	male	GCB	IV	B	3	4	PR	SD	16	dead
RR019	DL22	Yes	43	male	non_GCB	IV	A	3	4	PR	PD	25	dead
RR020	DL60	Yes	34	male	non_GCB	IV	B	3	4	PD	PD	31	dead
RR021	DL61	Yes	67	male	GCB	IV	B	4	4	PD	PR	36	dead
RR022	DL62	Yes	69	male	non_GCB	IV	B	1	4	PD	CR	35	dead
RR023	DL30	No	28	male	non_GCB	IV	A	2	3	PD	PD	7	dead
RR024	DL39	No	64	female	GCB	IV	A	3	5	PR	PD	10	dead
RR026	DL63	Yes	37	male	non_GCB	IV	A	1	3	CR	CR	57	survive
RR027	DL64	Yes	31	male	GCB	IV	B	3	4	CR	PD	14	dead
RR028	DL65	Yes	50	female	GCB	IV	B	3	4	PD	PD	15	dead
RR029	DL25	Yes	49	female	GCB	III	A	1	2	PR	PD	27	dead
RR030	DL24	Yes	65	male	non_GCB	IV	B	1	4	PR	PD	16	dead
RR031	DL52	Yes	56	male	non_GCB	II	A	1	1	CR	CR	38	dead
RR032	DL7	Yes	64	female	non_GCB	IV	A	2	5	CR	PD	40	dead
RR033	DL93	Yes	31	female	GCB	IV	A	1	3	PD	PD	17	dead
RR034	DL91	No	66	female	non_GCB	II	A	1	1	PD	PR	9	dead
RR035	DL66	Yes	62	female	non_GCB	IV	B	1	4	PR	PD	32	dead
RR036	DL42	Yes	41	female	non_GCB	IV	A	1	3	PR	PD	44	survive
RR037	DL67	Yes	33	female	GCB	IV	B	1	2	CR	CR	39	dead
RR038	DL87	Yes	65	female	non_GCB	II	B	1	2	SD	SD	16	dead
RR039	DL95	No	37	female	non_GCB	IV	B	1	2	PR	CR	34	dead
RR041	DL43	Yes	51	male	non_GCB	IV	B	1	2	PD	PD	36	dead
RR042	DL68	Yes	30	male	non_GCB	IV	B	3	4	SD	PD	9	dead
RR043	DL26	Yes	26	male	non_GCB	IV	A	1	3	PD	PR	29	dead
RR044	DL86	Yes	38	male	non_GCB	I	A	1	1	SD	PR	29	dead
RR045	DL21	Yes	67	male	non_GCB	IV	B	1	4	PR	PD	14	dead
RR046	DL15	Yes	55	female	non_GCB	IV	B	1	2	PR	PR	65	survive
RR047	DL44	Yes	39	male	non_GCB	IV	B	1	3	PR	PD	49	survive
RR048	DL17	Yes	38	male	GCB	IV	B	4	4	CR	PD	12	dead
RR050	DL96	No	45	female	non_GCB	IV	A	1	3	CR	CR	69	survive
RR052	DL92	Yes	70	female	non_GCB	IV	B	1	4	PD	CR	34	dead
RR053	DL27	Yes	47	female	GCB	IV	A	1	3	SD	PD	31	dead
RR054	DL79	No	70	male	non_GCB	IV	A	2	4	PR	PR	17	dead
RR055	DL8	Yes	71	female	non_GCB	IV	B	3	5	CR	CR	18	dead
RR056	DL5	Yes	48	female	non_GCB	IV	B	4	3	PD	PD	19	dead

RR057	DL69	Yes	43	male	GCB	IV	B	1	3	PD	PR	na	na
RR058	DL70	Yes	38	male	GCB	III	A	1	2	PR	PR	28	dead
RR061	DL45	Yes	65	female	non_GCB	IV	B	2	4	PR	PD	18	dead
RR062	DL10	No	35	female	GCB	IV	B	1	3	CR	SD	15	dead
RR063	DL72	Yes	31	male	non_GCB	IV	B	4	4	CR	PR	18	dead
RR064	DL29	Yes	34	male	non_GCB	IV	A	2	3	PR	PR	32	dead
RR065	DL14	Yes	47	female	GCB	IV	A	2	3	PR	PR	69	survive
RR066	DL73	Yes	28	female	non_GCB	IV	B	4	4	CR	PD	4	dead
RR067	DL46	Yes	56	male	non_GCB	IV	A	1	2	PD	PR	38	dead
RR068	DL74	Yes	44	male	GCB	IV	B	1	3	CR	PR	31	dead
RR069	DL90	Yes	63	female	GCB	IV	B	3	5	PR	PR	23	dead
RR070	DL4	No	55	male	non_GCB	IV	A	3	3	PD	SD	9	dead
RR071	DL75	Yes	60	female	GCB	I	A	1	2	PR	PR	39	dead
RR072	DL81	No	37	female	GCB	IV	A	1	3	PR	CR	33	dead
RR073	DL9	Yes	53	male	non_GCB	II	B	1	1	PR	PR	7	dead
RR074	DL16	Yes	29	female	GCB	IV	B	1	3	PR	PD	17	dead
RR077	DL18	Yes	44	male	non_GCB	IV	B	1	2	CR	PD	63	survive
RR078	DL76	Yes	53	male	GCB	III	A	1	3	PR	PR	27	dead
RR079	DL13	Yes	35	male	GCB	IV	B	1	2	PD	PD	11	dead
RR081	DL47	Yes	68	female	non_GCB	IV	B	3	5	PD	PR	14	dead
RR082	DL82	Yes	60	female	non_GCB	IV	B	3	4	CR	CR	61	survive
RR083	DL12	No	39	male	GCB	II	A	1	2	PD	SD	16	dead
RR084	DL77	Yes	50	female	GCB	IV	A	4	3	CR	PD	31	dead
RR085	DL28	Yes	31	female	non_GCB	IV	B	2	3	SD	PR	23	dead
RR086	DL48	Yes	72	male	non_GCB	IV	A	2	4	PR	PR	52	survive
RR088	DL2	Yes	40	male	non_GCB	IV	A	1	3	CR	PR	55	survive
RR089	DL6	No	66	female	non_GCB	IV	A	3	5	PR	PD	8	dead
RR090	DL37	Yes	66	female	non_GCB	III	A	2	2	PR	PR	51	survive
RR091	DL50	No	45	female	non_GCB	IV	B	1	3	PR	PD	24	dead
RR092	DL19	Yes	61	male	non_GCB	IV	A	2	4	CR	PD	37	dead
RR093	DL33	Yes	49	male	GCB	I	A	1	1	PR	PD	54	survive
RR094	DL84	No	48	female	non_GCB	II	A	1	1	PD	PR	27	dead
RR095	DL89	Yes	51	male	non_GCB	IV	B	3	4	CR	PR	21	dead
RR096	DL85	No	77	female	GCB	IV	A	1	3	PD	PR	8	dead
