# Supplementary figures and images for: Characteristics and prognosis of rrDLBCL with TP53 mutations and a high‐risk subgroup represented by the co‐mutations of DDX3X‐TP53
Source: Cancer Med. 2023 Mar 27;12(9):10267–79. doi: 10.1002/cam4.5756 (PMC10225230; doi:10.1002/cam4.5756)

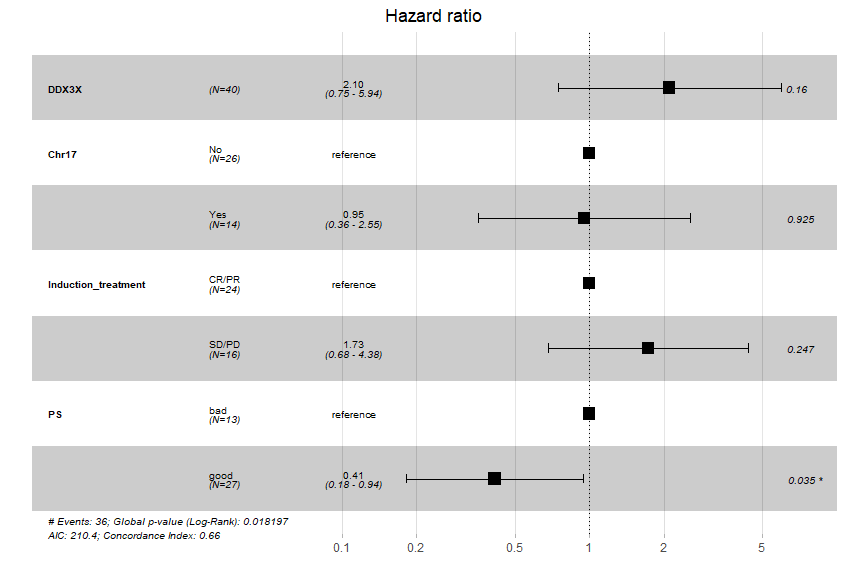

Supplement: Supplementary file 4 — Figure S1 [file CAM4-12-10267-s006.tiff]

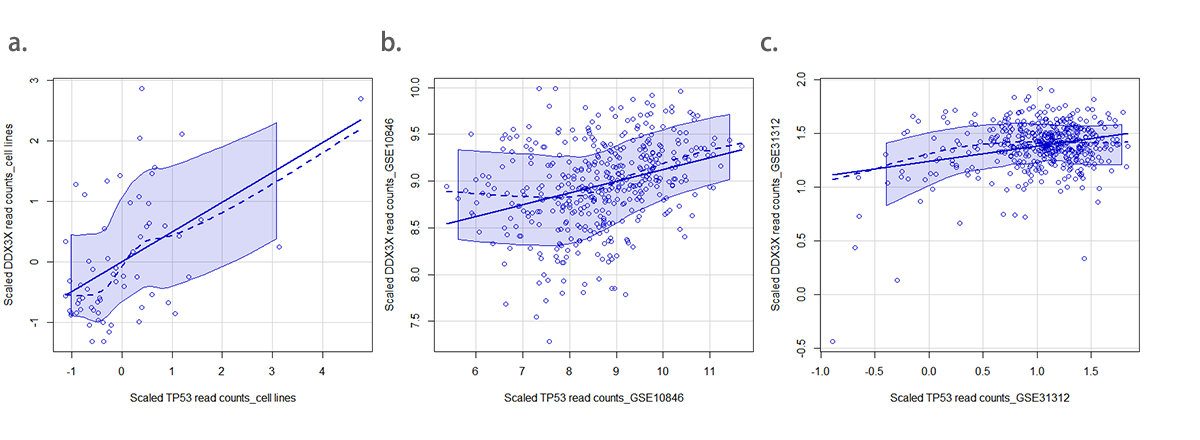

Supplement: Supplementary file 5 — Figure S2 [file CAM4-12-10267-s004.png]
